# Characterization of anisotropic turbulence behavior in pulsatile blood flow

**DOI:** 10.1007/s10237-020-01396-3

**Published:** 2020-10-22

**Authors:** Magnus Andersson, Matts Karlsson

**Affiliations:** Department of Management and Engineering, Linköping University, SE-581 83 Linköping, Sweden

**Keywords:** Barycentric anisotropy invariant map, Patient-specific scale-resolved computational hemodynamics, Reynolds stress and dissipation tensor, MRI turbulence measurements, Verification and validation

## Abstract

Turbulent-like hemodynamics with prominent cycle-to-cycle flow variations have received increased attention as a potential stimulus for cardiovascular diseases. These turbulent conditions are typically evaluated in a statistical sense from single scalars extracted from ensemble-averaged tensors (such as the Reynolds stress tensor), limiting the amount of information that can be used for physical interpretations and quality assessments of numerical models. In this study, barycentric anisotropy invariant mapping was used to demonstrate an efficient and comprehensive approach to characterize turbulence-related tensor fields in patient-specific cardiovascular flows, obtained from scale-resolving large eddy simulations. These techniques were also used to analyze some common modeling compromises as well as MRI turbulence measurements through an idealized constriction. The proposed method found explicit sites of elevated turbulence anisotropy, including a broad but time-varying spectrum of characteristics over the flow deceleration phase, which was different for both the steady inflow and Reynolds-averaged Navier–Stokes modeling assumptions. Qualitatively, the MRI results showed overall expected post-stenotic turbulence characteristics, however, also with apparent regions of unrealizable or conceivably physically unrealistic conditions, including the highest turbulence intensity ranges. These findings suggest that more detailed studies of MRI-measured turbulence fields are needed, which hopefully can be assisted by more comprehensive evaluation tools such as the once described herein.

## Introduction

Turbulence exhibits a wide range of cascading eddies, from the largest energy-containing macro-structures (integral length scale $\sim$ geometry) down to the smallest microscale whorls (Kolmogorov scales $\sim$ few tens of microns in blood flow) (Antiga and Steinman 2009), where the energy is dissipated into heat. The local ensemble of these eddies will reflect on the level of velocity fluctuations and turbulence-related momentum transport in different directions, which is highly affected by the gradients of the main flow. From a time-averaged point of view, these characteristics will favor specific axes of dependence and independence where the turbulence activity is strong or weak, respectively (Banerjee et al. 2007).

Turbulent-like hemodynamics have received increased attention in recent years as a phenotypic marker, suggested by the growing number of publications on the topic, with a diverse presence at different cardiovascular sites. Turbulence drains energy from the bloodstream, which increases the pressure losses and promotes higher risks of blood damage (hemolysis and platelet activation) as a result of elevated shear stresses and energy dissipation on the cellular level (Antiga and Steinman 2009; Morshed et al. 2014). Turbulence characteristics are also known to increase the susceptibility and progression of several vascular diseases (Chiu and Chien 2011; Kwak et al. 2014; Cunnane et al. 2017). The prediction of turbulence-related descriptors is an ongoing endeavor in the research community, where 4D Flow MRI (three-dimensional, time-resolved, phase-contrast magnetic resonance imaging) measurements and CFD (computational fluid dynamics) simulations play a dominating role. Both these disciplines are reliant on well-verified techniques that can provide reliable results, which imply the need for sufficient validation methods and uncertainty estimates (Steinman and Migliavacca 2018)—not least for the turbulence regimes, which are governed by much more complex flow physics that cannot easily be described by a few scalar quantities, in contrast to laminar physiological conditions.

A common way to quantify these turbulent properties is to consider the Reynolds stress tensor, which describes the statistical ensemble-average correlation between the fluctuating velocity components (or mean momentum flux due to the turbulent motion) in the coordinate directions, i.e., six independent stresses. Unfortunately, only limited knowledge can be gained by assessing the magnitude of these stresses alone in patient-specific flows, as the coordinate axes are oriented arbitrarily. Even in cases where the flow is aligned with the geometry, the overall characteristics of the turbulence field would be difficult to interpret without consider considering more than one scalar quantity (Banerjee et al. 2007).

**Fig. 1:**
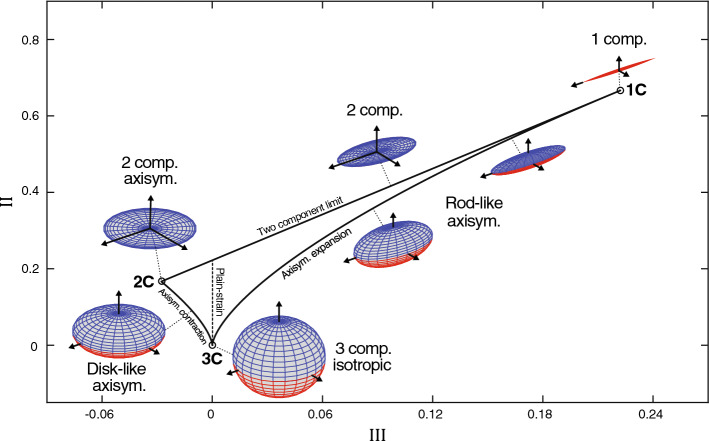
Turbulence states characterized by the anisotropy tensor in principal invariant coordinates. All physically realizable states of turbulence are constrained to a limited region, called the anisotropy invariant map (AIM) or Lumley triangle, and describe the relative size of the eigenvalues along the orthogonal principal axes (see glyph examples), i.e., the turbulence componentiality. The AIM corners bound three primary states: one-component (1C), two-component axisymmetric (2C), and three-component isotropic (3C) turbulence. These states are joint by different boundaries: axisymmetric expansion (rod-like turbulence), axisymmetric contraction (disk-like turbulence), and the two-component limit (pancake-like turbulence). Along the plain-strain region, one anisotropy eigenvalue is zero, whereby turbulence only commutes in planes. Reprinted from Andersson et al. (2019), with permission from Elsevier

**Fig. 2:**
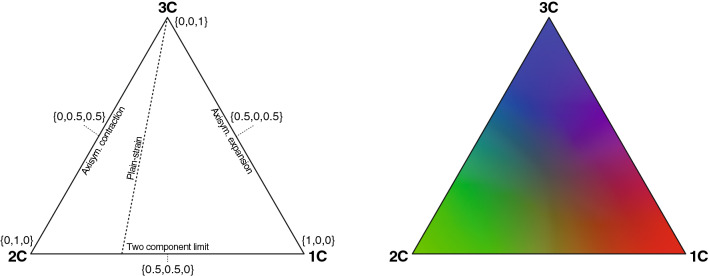
Barycentric colormap of turbulence anisotropy. With barycentric mapping all limiting states are connected by lines, in contrast to the nonlinear Lumley triangle (Fig. 1), forming a equilateral triangle. The local states are governed by the combined weights $\{C_{1C},C_{2C},C_{3C}\}$, i.e., the anisotropy tensor coordinates, which individually scales from 1 to 0, from the limiting state to the opposite corner, respectively. By associating color triplets to these weights, e.g., red, green, and blue, each realizable state in the barycentric map can be represented by a specific color. Further details are given in Fig. 1

To obtain a more complete picture of the turbulence behavior, both the tensor (principal) invariants and eigenvectors can be taking into account, which can provide single-point information such as amplitude, shape (anisotropy), and orientation of the turbulence stresses. The first principal invariant (the trace) gives an estimate of the turbulence magnitude. The anisotropy-related behavior can be extracted by only considering the deviatoric (traceless) part of the Reynolds stress tensor. These anisotropic characteristics can be featured in a so-called anisotropy invariant map (AIM) or Lumley triangle (Fig. 1) (Lumley and Newman 1977), which describes the relative strength of the velocity fluctuations in the principal coordinate axes, often referred to as the turbulence componentality (Helgeland et al. 2004). AIMs can be used to define the degree and nature of the turbulence anisotropy for any given symmetric second-order tensor, e.g., related to the mean strain rate and dissipation rate tensor (Banerjee et al. 2007). Since the introduction of the Lumley triangle, more intuitive representation of the AIM has been developed, including barycentric mapping (Banerjee et al. 2007) that provides a more interpretable equilateral triangle, and recently with the addition of point-specific color triplets (Fig. 2) (Emory and Iaccarino 2014), which can be used to visualize the turbulence states directly in the physical domain.

The utility of AIMs have been reported for a diverse set of flow applications (Banerjee et al. 2007; Emory and Iaccarino 2014), e.g., for physical interpretation of turbulence stresses (Kassinos et al. 2001; Choi and Lumley 2001; Andersson et al. 2015) as well as turbulence model development and comparison (Liu and Pletcher 2008; Philips et al. 2011; Banerjee et al. 2009), while no studies have so far been found in the biofluid community. Quantification of the anisotropic turbulence behavior may provide deeper insights into the physiological/clinical relevance of these conditions in various patient-specific flows and complement traditional hemodynamic descriptors. These characterization techniques could also be valuable assets for evaluating CFD models as well as MRI measurement strategies. In computational hemodynamics, the validity of some common modeling strategies could be investigated, such as choosing a steady flow regime versus a more realistic pulsatile condition, or performing turbulence modeling using a RANS (Reynolds-averaged Navier–Stokes) approach versus a scale-resolving method like LES (large eddy simulations), or the choice of using no explicit turbulence model, so-called coarse DNS (direct numerical simulations). For tensor-based MRI turbulence measurements (Haraldsson et al. 2015; Kefayati et al. 2015), Reynolds-stress-related quantities have been used with the goal to improve noninvasive predictions of pressure losses (Ha et al. 2017, 2019) and susceptibility to blood damage (Ha et al. 2016). The robustness and reliability of these predictors are governed by representable tensor properties. Here, barycentric anisotropy mapping could test the validity of these turbulence measurements, e.g., in a controlled environment against well-resolved CFD reference data, while also assessing the data realizability. With unfavorable acquisition strategies as well as improper data aggregation, certain points may fall outside the physical space of the AIM, as shown in this study.

This background has emphasized on the importance of exploring a more broadband description of turbulence-related tensor characteristic in hemodynamic-related applications. Reliable predictions of these turbulence fields in different disciplines require adequate verification and validation practices. The present study aimed to demonstrate the potential utility of these visualization techniques in different areas touched above. In particular, we explored the magnitude and anisotropic behavior of the Reynolds stress and turbulence dissipation tensor in the turbulent region of a patient-specific LES model of an aortic coarctation (CoA). These results were thereafter used as a reference to evaluate the impact of different CFD modeling strategies (steady inflow and RANS model). The same techniques were also used to evaluate MRI-based turbulence measurements in different post-orifice flows through a straight pipe.

## Methods

In this section, a brief description of the utilized techniques and methods is outlined. More details regarding the MRI acquisition, segmentation, modeling assumptions, and limitations can be found in previous studies and supplementary materials within Andersson et al. (2015, 2017, 2019).

### Turbulence anisotropy

Reynolds stresses are based on the ensemble-averaged correlation between two fluctuating velocity signals, which in pulsatile flows can be extracted from the cycle-to-cycle flow variations. In this study, the operators used to define the anisotropy tensors, and related parameters are given in Table 1. A triple decomposition technique (Hussain and Reynolds 1970) was used to separate the velocity field from its mean, cyclic, and turbulence-related parts (Table 1, Eq. 1). The phase-averaged velocities were estimated by calculating the individual grid points mean value at a predetermined set of phase positions sampled over a desirable window of cardiac cycles (Eq. 2). The Reynolds stress tensor ${R}_{ij}$ and corresponding dissipation rate tensor $\epsilon _{ij}$ were thereafter constructed by calculating the phase-averaged correlation between the six independent combinations of the fluctuating velocity signal (Eq. 3) and its spatial gradients (Eq. 4), respectively. The tensor magnitude was defined by the turbulent kinetic energy (TKE, *k*) and its dissipation rate $\epsilon$ (Eqs. 5 and 6, respectively), i.e., half the trace, and thereafter removed to construct the anisotropy (traceless) part of the Reynolds stress tensor ${b}_{ij}$ and dissipation tensor ${d}_{ij}$ (Eqs. 7 and 8, respectively). These normalized anisotropy tensors are real and symmetric (${a}_{ij}$=${a}_{ji}$), meaning that they can be diagonalized by an orthonormal matrix according to the spectral theorem (Eq. 9), providing orthogonal eigenvectors along the principal axes and real eigenvalues $\lambda _{l}$. By rearranging the eigenvalues, the anisotropy tensor can be represented in the unique (invariant) canonical form (Eq. 10). The second (${\text {II}}$) and third principal invariant (${\text {III}}$) of the anisotropy tensor can be associated to the degree and nature of the turbulence anisotropy (Eqs. 11 and 12, respectively), which together can establish a bounded region referred to as an anisotropy invariant map (Fig. 1) or sometimes the Lumley triangle (Lumley and Newman 1977). Within this map, all physically realizable states of the turbulence can be found and is attained when the tensor components and determinant are constrained as (Schumann 1977)

$$\begin{aligned}&{a}_{\alpha \alpha }\geqslant 0,\quad {a}_{\alpha \alpha }+{a}_{\beta \beta }\geqslant 2|{a}_{\alpha \beta }|,\quad \\&\quad {\text {det}}({a}_{ij})\geqslant 0,\quad \alpha ,\beta =\left\{ 1,2,3\right\} . \end{aligned}$$

where no summation is applied for the Greek indices. These invariants are nonlinear functions of the anisotropy tensor eigenvalues $\lambda _{i}$ that represent the relative strength of the different fluctuating components, often referred to as turbulence componentiality (Helgeland et al. 2004). It is important to note that the eigenvector’s directionality is identical between the non-normalized tensor and the corresponding anisotropy tensor, while the eigenvalues are scaled. For example, the eigenvalue relation between the Reynolds stress tensor ${R}_{ij}$ and corresponding anisotropy tensor ${b}_{ij}$ are given by $\lambda _{i}=\lambda ^{R}_{i}/2k-1/3$.

The corners of the AIM can be described by three fundamental limiting turbulence states (Fig. 1):

- *One-component turbulence (1C).* Here the tensor only have one nonzero eigenvalue ($\lambda ^{R}_{i}\ne 0$)^1^. Anisotropy eigenvalues are $\left\{ \lambda _{1},\lambda _{2},\lambda _{3}\right\} \!=\!$ $\left\{ 2/3,-1/3,-1/3\right\}$, where the turbulence is only acting in one direction along the nonzero eigenvector.
- *Two-component axisymmetric turbulence (2C).* Here the tensor have two nonzero eigenvalues of equal size. Anisotropy eigenvalues are $\left\{ \lambda _{1},\lambda _{2},\lambda _{3}\right\} \!=\! \left\{ 1/6,1/6,-1/3\right\}$, where one direction is inactive and turbulence only acts uniformly in a plane.
- *Three-component isotropic turbulence (3C).* Here the tensor have three nonzero eigenvalues of equal size. Anisotropy eigenvalues are $\left\{ \lambda _{1},\lambda _{2},\lambda _{3}\right\} \!=\! \left\{ 0,0,0\right\}$. This is the pure isotropic state where turbulent fluctuations acts randomly in all directions.

The borders between these limiting states are described by a mix of intermediate characteristics:

- *Two-component limit.* Represented by ellipse-like (pancake-shaped) turbulence and $\lambda _{1}\!=\!\lambda _{2}\gg \lambda _{3}$
- *Axisymmetric expansion.* Represented by rod-like (cigar-shaped) turbulence and $\lambda _{1}\gg \lambda _{2}\!=\!\lambda _{3}$
- *Axisymmetric contraction.* Represented by disc-like (oblate spheroid) turbulence and $\lambda _{1}\!=\!\lambda _{2}\gg \lambda _{3}$

Across the AIM a special turbulence state can be found for ${\text {III}}\!=\!0$ called the *plain-strain* limit. Along this line one anisotropy eigenvalue $\lambda _{i}$ is zero (except at 3C corner, where, e.g., all $\lambda _{i}\!=\!0$ and $\lambda ^{R}_{i}\!=\!2k/3$ ) and the mean momentum exchange due to turbulent fluctuating only occurs along a plane, while the third principal direction is governed by isotropic fluctuations that does not contribute to any momentum transport.

#### Barycentric AIM

A drawback with the Lumley triangle is its nonlinear features, which distort these results unevenly across the map and make interpretations less intuitive. This deficiency was removed by proposing a convex (linear) combination of the three limiting states (1C, 2C, and 3C) in barycentric coordinates (Banerjee et al. 2007). From the spectral theorem, the anisotropy tensor can be decomposed into three tensor basis $\left\{ \widetilde{a}_{1C}, \widetilde{a}_{2C}, \widetilde{a}_{3C}\right\}$, each representing the canonical eigenvalue matrix of, respectively, limiting states (Eq. 13). By also introducing eigenvalue-related weights $\left\{ C_{1C}, C_{2C}, C_{3C}\right\}$ to each basis, with sophisticated constraints, a linear deviation measure from each limiting state can be formed. In fact, these weights control the coordinates in the barycentric map (Eq. 14), in the range of [0, 1], and determines the contribution from each extreme state that together specifies the overall turbulence anisotropy characteristics (Fig. 2). The linear nature of this map also makes it easy to define a scalar that estimates the departure from the isotropic state (3C), or degree of anisotropy herein referred to as the anisotropy index (*AI*) (Eq. 15), as well as interpretation of deviation measures (Eq. 16).

A general disadvantage with AIMs is the difficulty to highlight the turbulence anisotropy on a broader scale in the physical domain, with analysis typically limited to a trajectory of points along the region of interest. This lack of spatial context was recently circumvented by simply using the barycentric map as a color triangle (Emory and Iaccarino 2014), where three primary colors are associated with the limiting turbulence states. This approach could directly visualize the anisotropy characteristics in the domain (e.g., over a plane) and are not limited to large amounts of data. The barycentric colormap is easily formed by multiplying each color triplet by the corresponding weights. In this study, RGB color triplets were used to represent the componentality contours (Eq. 17), with 1C=red, 2C=green, and 3C=blue (Fig. 2). Also, these colormaps can easily be manipulated to, e.g., bring more contrast to specific states within the AIM (Emory and Iaccarino 2014).

**Table 1:** Parameter definitions

Operators and parameters	Definitions		Notations
Triple decomposition	${u_i}\left( {\mathbf {x}},t\right) = \overline{u}_i\left( {\mathbf {x}}\right) + \widetilde{u}_i\left( {\mathbf {x}},t\right) + {u^{\prime }_{i}}\left( {\mathbf {x}},t\right)$	(1)	${\mathbf{x}} (x,y,z)$
Phase averaging	$\langle {u_i}\rangle \left( {\mathbf {x}},t\right) = \overline{u}_i\left({ \mathbf {x}}\right) + \widetilde{u}_i\left( {\mathbf {x}},t\right) =\frac{1}{N}\sum \limits _{n=0}^{N-1}{u_i}\left( {\mathbf {x}},t+nT\right)$	(2)	$u^{\prime }_{i}={u}_i-\langle {u_i}\rangle$
Reynolds stress tensor	${R}_{ij} =\rho \langle {u^{\prime }_{i}}{u^{\prime }_{j}}\rangle =\frac{\rho }{N}\sum \limits _{n=0}^{N-1}{u^{\prime }_{i}}{u^{\prime }_{j}}$	(3)	$3\times 3$ sym.
Dissipation rate of ${R}_{ij}$	$\epsilon _{ij} = \langle 2{\mu (u^{\prime }_{i,k}}{u^{\prime }_{j,k})}\rangle =\frac{2}{N}\sum \limits _{n=0}^{N-1}{\mu (u^{\prime }_{i,k}}{u^{\prime }_{j,k})}$	(4)	$3\times 3$ sym.
Turbulence kinetic energy	$k=\tfrac{1}{2}{R}_{kk}$	(5)	[Pa]
Dissipation rate of *k*	$\epsilon =\tfrac{1}{2}{\epsilon }_{kk}$	(6)	[Pa s$^{-1}$]
Anisotropy tensors	${b}_{ij}={R}_{ij}/2k-\delta _{ij}/3$	(7)	${b}_{kk}=0$
	${d}_{ij}={\epsilon }_{ij}/2\epsilon -\delta _{ij}/3$	(8)	${{d}_{kk}}=0$
Spectral decomposition theorem	${a}_{ij}=v_{ik}\Lambda _{kl}v_{jl}$	(9)	$\in \lambda _l[-\tfrac{1}{3}, \tfrac{2}{3}]$
${a}_{ij}$ on canonical form	$\widetilde{a}_{ij}=\lambda _i\delta _{ij}=\lambda _1 x_{1}x^{T}_{1}+\lambda _2 x_{2}x^{T}_{2}+\lambda _3 x_{3}x^{T}_{3}$	(10)	$\lambda _1\geqslant \lambda _2\geqslant \lambda _3$
2nd principal invariant	$\text {II}={a}_{ij}{a}_{ji}=2(\lambda ^2_1+\lambda _1\lambda _2+\lambda ^2_2)$	(11)	$\in [0, \tfrac{2}{3}]$
3rd principal invariant	$\text {III}={a}_{ij}{a}_{jk}{a}_{ki}=-3\lambda _1\lambda _2(\lambda _1+\lambda _2)$	(12)	$\in [-\tfrac{1}{36}, \tfrac{2}{9}]$
Convex merge of the limiting states	$$\begin{array}{l} \widetilde{a}_{ij}=C_{1C}\widetilde{a}_{1C}+C_{2C}\widetilde{a}_{2C}+C_{3C}\widetilde{a}_{3C}, \\ \widetilde{a}_{1C}=diag\left\{ 2/3,-1/3,-1/3\right\} , \\ \widetilde{a}_{2C}=diag\left\{ 1/6,1/6,-1/3\right\} , \\ \widetilde{a}_{3C}=diag\left\{ 0,0,0\right\} , \\ \left\{ C_{1C}, C_{2C}, C_{3C}\right\} =\left\{ \lambda _1-\lambda _2, 2(\lambda _2-\lambda _3), 3\lambda _3+1\right\} \end{array}$$	(13)	$$\begin{array}{c}\in C_{iC}[0, 1], \\ \sum C_{iC}=1 \end{array}$$
Barycentric coordinates	$$\begin{array}{l} x_B=C_{1C}x_{1C}+C_{2C}x_{2C}+C_{3C}x_{3C} \\ y_B=C_{1C}y_{1C}+C_{2C}y_{2C}+C_{3C}y_{3C} \end{array}$$	(14)	$$\begin{array}{l} \in [0, 1], \\ \in [0, \sqrt{3}/2] \end{array}$$
Anisotropy index	$AI=C_{1C}+C_{2C}$	(15)	$\in [0, 1]$
Root–mean–square deviation	$C_{{\mathrm{rms}}}=\Big (\frac{1}{3}\sum \limits _{i=1}^{3}(\bigtriangleup C_{iC})^2\Big )^{\frac{1}{2}}$	(16)	$\in [0, 1]$
Barycentric color triplets	$[{\mathrm{R}}~{\mathrm{G}}~{\mathrm{B}}]^T=C_{1C}\underbrace{[1~0~0]^T}_\text {red}+C_{2C}\underbrace{[0~1~0]^T}_\text {green}+C_{3C}\underbrace{[0~0~1]^T}_\text {blue}$	(17)	$\in [0, 1]$

Symbols: $u_i=$ velocity components, $\overline{u}_i=$ mean value, $\widetilde{u}_i=$ cycle-related variations, $u^{\prime }_{i}=$ turbulence-related stochastic fluctuations, $\langle u_i\rangle =$ phase-averaged, $N=$ number of cardiac cycles, $T=$ constant cardiac period, $t=$ time in the cardiac cycle, $\mu =$ dynamic viscosity, $\rho =$ fluid density (1060 kg m$^{-3}$), $\delta _{ij}=$ Kronecker delta, ${a}_{ij}=$ normalized symmetric 2nd-order anisotropy tensor (e.g., ${b}_{ij}$ or ${d}_{ij}$), $v_{ij}=$ matrix of orthonormal eigenvectors, $\Lambda _{kl}=$ traceless diagonal matrix of eigenvalues $\lambda _l$. $\left\{ x_{1},x_{2},x_{3}\right\} =$ eigenvectors corresponding to the reordered eigenvalues $\lambda _i\delta _{ij}=diag\left\{ \lambda _{max}=\lambda _{1},\lambda _{int}=\lambda _{2}, \lambda _{min}=\lambda _{3}\right\}$, $C_{iC}=$ anisotropy tensor coordinates (weights) in the limiting states tensor basis ($\widetilde{a}_{1C},\widetilde{a}_{2C},\widetilde{a}_{3C}$), where $\bigtriangleup C_{iC}$ represents a deviation measure between two cases

### Patient-specific CFD model

The patient-specific CFD model of the aortic coarctation and surrounding vessels were derived from MRI measurements during resting conditions, where appropriate consents from the patient and local ethics committee were given. The freely available software Segment (Heiberg et al. 2010) was used to extract a stereolithography representation of the luminal boundary and supracoronary inflow condition. The peak-to-peak trans-stenotic pressure drop was measured to 22 mmHg, which is commonly viewed as significant for intervention (Turner and Gaines 2007). The wall was assumed rigid with a no-slip condition. A flow rate waveform was assigned to the inlet of the ascending aorta (AAo), assuming a flat velocity profile, while the flow rate out of each aortic arc branch was weighted by a square law (Zamir et al. 1992). A static pressure boundary condition was assigned to the outlet of the descending aorta (DAo). A non-Newtonian blood model governed the fluid rheology (Carreau 1972; Cho and Kensey 1991), as shear-thinning fluid properties have shown to slightly delay turbulence transition and impose turbulence dampening effects (Biswas et al. 2016; Khan et al. 2019). Large eddy simulation was used to resolve the energy-containing scales of the flow, while the effect from the subgrid scales flow was represented by the WALE (wall-adaptive local eddy-viscosity) model (Nicoud and Ducros 1999). The domain was reconstructed by 6 million cells (MC) using a hexahedral O-grid approach in ANSYS ICEM CFD 15.0 (ANSYS Inc, Canonsburg, PA, USA). Adaptive time-stepping was used to maintaining the Courant–Friedrichs–Lewy number below unity. Fifty (50) cardiac cycles were used for phase averaging, which was seen as the upper limit regarding computational costs ($\sim$500k CPU hours on the local supercomputer). Adding more cycles has previously been shown to have a minor effect on the overall amount of TKE (Andersson et al. 2015) and wall shear stress characteristics (Andersson et al. 2017). Five cardiac cycles were simulated before the phase averaging to minimize initialization effects on the flow. Data were saved every 0.01 s during each cardiac cycle (T = 1 s), which was considered sufficient to capture the essential flow features over the cycle.

The governing equations were solved using ANSYS CFX 15.0, a fully coupled, implicit finite volume solver. Temporal gradients were discretized by a second-order backward Euler scheme, the convective term by a central differencing scheme, and the diffusion and pressure gradient terms by tri-linear (finite element) shape functions. Rhie–Chow interpolation was used to assure local mass-conservation.

#### Simulation strategies

In patient-specific blood flow simulations, the pulsatile nature of the flow is sometimes ignored and simplified by a steady inflow condition, typically motivated by reduced computational costs, lack of sufficient boundary conditions, or representing a worst-case scenario. Comparison between these flow regimes has been performed on idealized flow conditions in pipes, revealing clear differences in turbulent flow behavior (Varghese et al. 2007, 2007; Manna et al. 2015). Taking into account further complexities in patient-specific cardiovascular flow models, a steady boundary condition assumption may not be a good reciprocal to mimic physiologically realistic flows. In this study, the validity of using a steady inflow was tested against the pulsatile reference model (Sect. 2.2). These steady inflows were set to 100% and 50% of the pulsatile peak flow rates, with the latter being close to the pulsatile mean flow rate, which are common assumptions. Time-averaged convergence of the Reynolds stresses was ensured by first removing any initialization effects and continued data sampling for 14 additional inlet-to-outlet flow-throughs. The remaining numerical procedures followed the reference model.

The increased availability of computational resources has open the doors for using scale-resolving simulations, such as LES, which in general are known to outperform simpler modeling strategies such as Reynolds-averaged turbulence models. RANS-based simulations are, however, still frequently used in cardiovascular applications, despite the discouragement to used these models to predict pulsatile, transitional, and relaminarizing types of flows (Mittal et al. 2003; Taylor and Steinman 2010). In LES, most energetic turbulent scales are resolved, while the impact from the smaller unresolved scales is modeled. In RANS simulations, the Reynolds stresses are modeled as a local function of the mean flow properties, with ad hoc assumptions and parameters tuned for universal flow cases. In this study, the commonly used *k–* $\omega$ based Shear–Stress–Transport (SST) model (Menter 1994) was compared to the baseline LES results.

The RANS simulation was executed in ANSYS CFX 19.1, using the 6 MC mesh with the same temporal scheme and resolution as the reference simulation. The convective term was evaluated with the High-Resolution scheme, which essentially is second-order accurate and ensures boundedness. In unsteady RANS (URANS) simulations of pulsatile flows, it is reasonable to assume that phase-averaged results are independent of the number of cardiac cycles as long as initialization effects have been removed. Here, the fourth cardiac cycle was therefore used for evaluation. The Reynolds stress anisotropy was obtained from the mean velocity gradients using the turbulent-viscosity hypothesis (Pope 2005).

### MRI measurements

The progressive development of 4D Flow MRI turbulence measurements have enabled better in vivo predictions of these conditions in different cardiovascular areas (Dyverfeldt et al. 2008; Hope et al. 2013; Dyverfeldt et al. 2015). The ICOSA6 (six-directional icosahedral) flow encoding scheme (Haraldsson et al. 2015; Kefayati et al. 2015) was initiated to measure all six components of the Reynolds stress tensor, from which improved estimates of pressure losses and shear-induced blood damage have been suggested (Ha et al. 2016, 2017, 2019). However, the general applicability of these techniques is highly dependent on knowing the accuracy of these predictors. In this investigation, anisotropy invariant mapping was used to evaluate these ICOSA6-based Reynolds stress characteristics measured in an experimental rig of a straight pipe (16 mm diameter) with a concentric orifice of 75$\%$ area reduction. The experimental rig was designed to produce fully developed laminar flow before the contraction. Two different steady flow rates were tested, corresponding to a Reynolds number of 2058 and 5383 in the large pipe section, respectively. Details regarding the experimental setup and MRI measurement procedures are given in a previous study (Ha et al. 2017). This analysis was limited to the evaluation of general and expected turbulence anisotropy characteristics observed in similar applications, as well as possible violation of the realizability constraints.

**Fig. 3:**
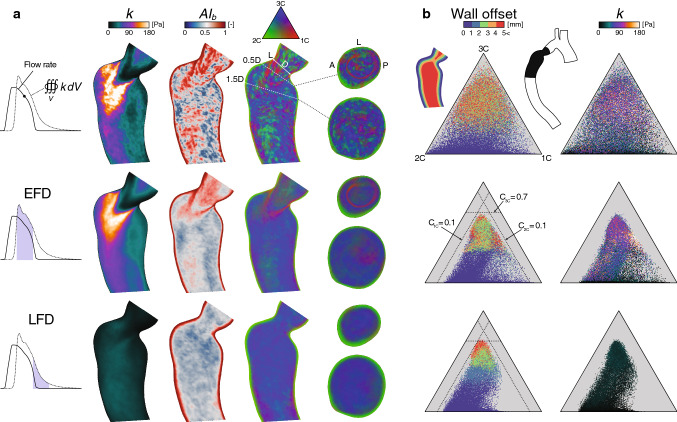
Patient-specific Reynolds stress characteristics. (**a** and **b**) Rows represent: a snapshot during the flow deceleration phase (top), time-averaged over the early (middle) and late (bottom) flow deceleration phase, denoted EFD and LFD, respectively. (**a**) Axial planes through the turbulent region (vessel inset in **b**, black region), colored by the turbulence kinetic energy (*k*), anisotropy index ($AI_b$), and barycentric map. Cross-sectional planes were added normal to the centerline at 0.5D and 1.5D downstream the smallest stenotic diameter (D). For reference, the left (L), anterior (A), and posterior (P) sides of the aorta were included. (**b**) Barycentric maps with 50k points extracted from the turbulence region (vessel inset, black region), colored by the wall-normal offset distance (left column), and *k* (right column). The seed points were randomly selected with an even spatial distribution. At EFD, the dashed lines demonstrate suggested borders of the, respectively, turbulent state, for reference also shown at LFD

**Fig. 4:**
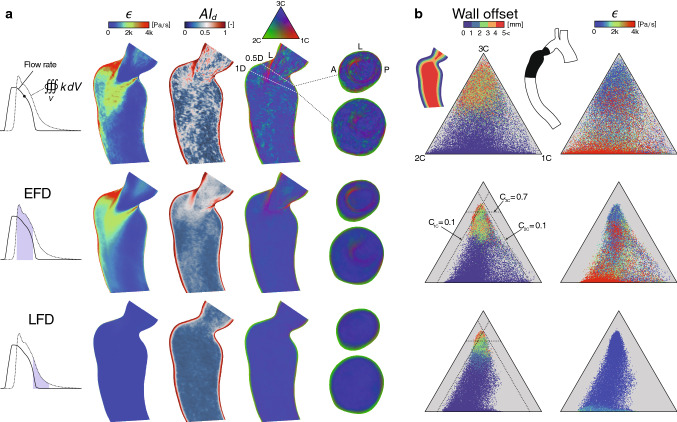
Patient-specific turbulence dissipation characteristics. (**a**) Axial planes colored by the dissipation rate of turbulence kinetic energy ($\epsilon$), anisotropy index ($AI_d$), and barycentric map with addition of cross-sectional planes at 0.5D and 1D. (**b**) Barycentric maps with 50k points, colored by the wall normal offset distance (left column) and $\epsilon$ (right column). Additional details are given in Fig. 3

## Results

**Fig. 5:**
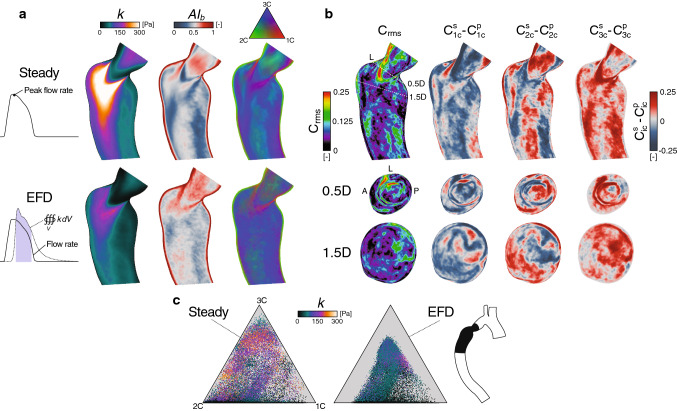
Steady versus pulsatile inflow condition effects on the Reynolds stress characteristics. (**a**) Axial planes colored by the turbulence kinetic energy (*k*), anisotropy index ($AI_b$), and barycentric AIM. (**b**) Deviation maps, showing the root–mean–square ($C_{rms}$, Eq. 16) and individual differences ($C^s_{ic}-C^p_{ic}$, superscripts: $s=$ steady, $p=$ pulsatile), of the AIM weights ranging from [0, 1]. (**c**) Barycentric maps with 50k points colored by *k*. Additional details are given in Fig. 3

**Fig. 6:**
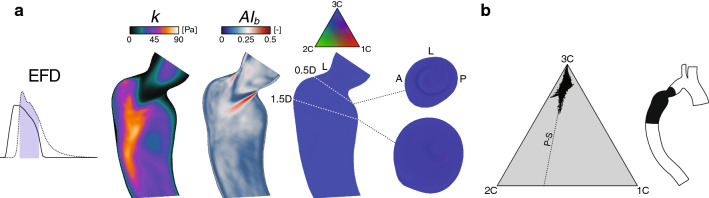
RANS-based Reynolds stress characteristics. (**a**) Axial planes colored by the turbulence kinetic energy (*k*), anisotropy index ($AI_b$) , and barycentric AIM with addition of cross-sectional planes at 0.5D and 1.5D. Note, $AI_b$ upper limit is set to 0.5. (**b**) Barycentric maps with 50k points. Additional details are given in Fig. 3

### Patient-specific turbulence anisotropy

The general patient-specific results herein are featured as either a phase-averaged snapshot (phase-instant) at the early flow deceleration phase, where the turbulence was most pronounced, or time-averaged over different flow stages in the cardiac cycles where a substantial shift in post-stenotic turbulent behavior could be noticed (Fig. 3a). The first stage was time-averaged over 20 ms in the early flow deceleration (EFD) phase, where a quasi-steady turbulent jet was formed and directed toward the opposing wall, resulting in the most intense turbulence field throughout the cycle. In the second flow stage, results were time-averaged over the subsequent 20 ms of the first stage, i.e., the late flow deceleration (LFD) phase. Here, loss of main flow momentum promoted jet breakup followed by gradual relaminarization. A more detailed view of these flow characteristics can be found in a previous work (Andersson et al. 2019). The anisotropy characteristics of the Reynolds stress and dissipation tensor were evaluated over axial and cross-sectional planes in the stenotic region (Figs. 3a and 4a) and over volumetric seed points projected into the barycentric map itself (Figs. 3b and 4b).

At EFD (Fig. 3a), the Reynolds stresses showed high anisotropy levels ($AI_b\!>\!0.5$), with a wide range of different turbulence states depicted by the barycentric map. In the near-wall region (within $\sim$1 mm wall offset), the stresses approach the two-component limit, as expected in the presence of a wall (Mansour et al. 1988). Away from the wall, the most elevated anisotropy occurred along the destabilizing shear-layers surrounding the jet, where the stress states had more weight toward the one-component limit (1C), and axisymmetric expansion bound, whereas the remaining region featured a variety of more three-component stress characteristics (Fig. 3b, wall offset). Overall, the phase-instant characteristics showed less coherent regions compared to the time-averaged results. At LFD, the aforementioned elevated 1C-like anisotropy was clearly absent, and the near-wall two-component layer slightly thickened. Within the maps (Fig. 3b), the phase-instant results revealed a much wider range of stress states in comparison with both time-averaged results, which showed distinct offsets from the suggested axisymmetric contraction ($C_{1C}=0.1$) and expansion ($C_{2C}=0.1$) borders, as well as the isotropic corner ($C_{3C}=0.7$). At LFD, a clear displacement could be noticed away from the 1C corner. The imposed TKE field showed elevated intensities ($k\!>\!$ 90 Pa) over a wide range of different stress states in the EFD phase, however, with a tendency of being moderate-to-low when approaching the 1C limit.

The turbulence dissipation rates (Fig. 4a) were overall high ($\epsilon \!>\!$ 2 kPa s$^{-1}$) along the turbulence intense post-stenotic jet and jet-opposing (left side) near-wall region of the aorta during EFD phase. Here, elevated anisotropy was found in the immediate jet vicinity, along the separated shear layers, with 1C-like characteristics, and, as expected, in the near-wall region. Compared to the Reynolds stresses, anisotropy boundary layer was slightly thinner, while the bulk flow generally entailed more isotropic characteristics. At LFD, the magnitude of the dissipation rates was substantially lower. In the phase-instant maps (Fig. 4b), higher dissipation rates could be associated with a fairly wide spectrum of different anisotropic states, with a majority of points located close to the two-component boundary. For time-averaged results, similar aforementioned bounding regions away from the axisymmetric contraction ($C_{1C}=0.1$) and expansion ($C_{2C}=0.1$) borders were found, although with a slight shift toward the 3C corner (close to $C_{3C}=0.8$).

### Steady versus pulsatile inflow

For the two investigated inflow rates, similar overall anisotropic Reynolds stress characteristics were noticed. Therefore, the 50% peak flow rate was excluded for brevity. The steady inflow results were compared with the time-averaged pulsatile results over the EFD phase (Fig. 5).

The steady condition clearly over-predicted the TKE magnitude before and after the coarctation (Fig. 5a). Areas of high and low anisotropy levels ($AI_b$) were fairly co-localized between the cases, but differed in magnitude. Also, a general regional agreement of the most extreme stress states could be observed, with, e.g., clearly 1C-dominant features in the vicinity of the turbulence jet. However, in the point-wise deviation analysis (Fig. 5b), both anisotropy weights ($C_{1C}$ and $C_{2C}$) showed over- as well as under-predicted regions in the order of 20%. The isotropy weight ($C_{3C}$), on the other hand, was overestimated for the steady inflow case in a substantial part of the turbulent region. These findings were also confirmed within the barycentric map (Fig. 5c), where the steady flow demonstrated a nearly full spectrum of stress states, in contrast to the time-averaged results over the EFD phase.

### RANS versus LES

The time-averaged RANS result showed little resemblance to the corresponding LES findings (Fig. 6), where both TKE and anisotropy levels were substantially under-predicted. In fact, the barycentric maps showed close to isotropic stress states throughout the turbulence domain, and only minor evidence of turbulence anisotropy could be seen in the vicinity of the plain-strain state. Furthermore, the RANS model failed to predict the high turbulence anisotropy expected in the near-wall region.

**Fig. 7:**
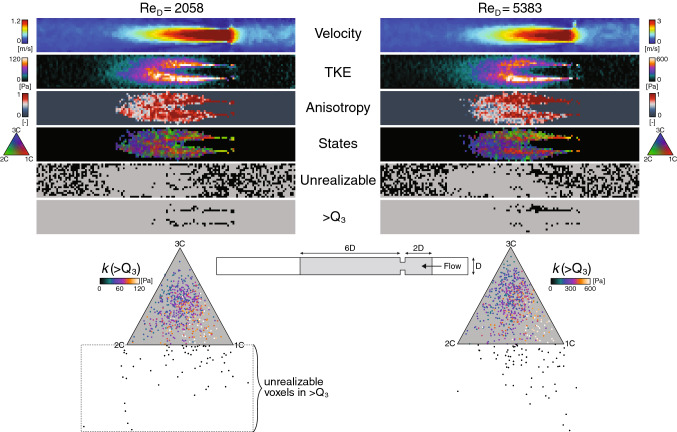
MRI-measured Reynolds stress characteristics in an idealized constriction. Axial symmetry planes (2D upstream to 6D downstream) at two different Reynolds numbers (${\text {Re}}_{\text {D}}$) based on the large diameter (D) of the pipe. Planes are colored by the velocity magnitude (Velocity), turbulence kinetic energy (TKE, *k*), anisotropy index (Anisotropy, $AI_b$), and barycentric AIM (States). Additional planes were added to show voxels that fall outside the AIM (black voxels), i.e., nonphysical turbulence states; considering all voxels (Unrealizable) and only the upper $25\%$ ($>\!Q_3$) of the post-orifice TKE values. The latter data range was also projected into the barycentric maps (bottom), which show the unrealizable voxels outside the triangular domain for both flow cases

### MRI measurements

In both measured cases, a clear turbulent jet could be seen immediately downstream of the orifice (Fig. 7), with near fivefold magnitude differences in the highest TKE ranges. Insubstantial parts of the destabilizing shear-layer surrounding the jet, expected areas of high turbulence anisotropy were found in both cases, while appearing more axisymmetric distributed within the pipe for the lowest flow rate. The barycentric map featured a wide range of different stress states in the turbulence spot, locally with somewhat dispersed (incoherent) patterns between neighboring voxels. Although some organized characteristics could be noticed in both flows, e.g., the 1C-like state along the expected shear-layer as well as more isotropic (3C-dominant) conditions in the more distal parts of the turbulent region. Overall, a large portion of unrealizable voxels was seen in the unfiltered data, especially in regions of low turbulence intensity and close to the wall, which may be expected due to increased noise level and partial volume effects. However, by only considering the data above the upper quartile range ($>\!Q_3$) of the TKE field, sizable regions of unrealizable voxels still prevailed. Quantitatively, these locations appeared to be co-localized with areas where elevated turbulence production may be expected. A clear low-to-high TKE magnitude trend was noticed within the barycentric maps when moving from the axisymmetric contraction boundary toward the 1C corner.

## Discussion

In this study, barycentric anisotropy invariant maps were used to showcase practical ways to characterize the turbulence-related tensor properties in patient-specific hemodynamics as well as MRI-measured flows, while also demonstrate the techniques potential utility to evaluate proper modeling and measurement strategies. The proposed method revealed distinct regions of elevated turbulence anisotropy of various characteristics in the post-stenotic flow, with evident differences between the steady inflow and RANS assumptions. For the MRI-assessed flows, some expected Reynolds stress anisotropy patterns were depicted, while also featuring a substantial degree of voxels with unrealizable characteristics.

### Patient-specific findings

Nowadays, turbulent-like hemodynamics are being more frequently observed by the refined CFD and measurement techniques, while its intrinsic pathophysiological role associated with cardiovascular diseases is still largely unknown. To narrow this gap, improved predictions and descriptors of these possible flow phenotypes are essential. Turbulent motions are highly irregular/chaotic and, therefore, typically treated from a statistical point-of-view by ensemble averaging the fluctuating flow variables over time. Here, the average change of momentum due to the turbulence environment, which locally is influenced by the superposition of velocity fluctuations generated by the convective movement of nearby eddies, is described by the second-moments within the Reynolds stress tensor. Due to the anisotropic nature of most real flows, the magnitude and orientation of these fluctuations will, in a time-averaged sense, conform to specific states, which can be characterized by the relative strength of the tensor eigenvectors. These fundamental principals also hold for the averaged dissipation rate of the Reynolds stresses (dissipation tensor). The main difference, however, is that Reynolds stresses are predominately influenced by the larger turbulence scales of the flow, whereas most of the dissipation rate occurs at much smaller scales where the turbulence field statistically are generally more isotropic and homogeneous.

The patient-specific results in this study showed clear evidence of anisotropy elevation for the Reynolds stress tensor $R_{ij}$ and turbulence dissipation rate tensor $\epsilon _{ij}$ (Figs. 3 and 4), with local magnitude and turbulence state differences. Within all time-averaged barycentric maps, a distinct retraction from the axisymmetric borders and 3C limiting state was observed, which was not depicted in the phase-instant results. Thus, the occurrence of these extreme stress states, in the margin of axisymmetric expansion and contraction boundaries, seems only to occur under a short time span as the main flow momentum decelerates. Qualitatively, both $R_{ij}$ and $\epsilon _{ij}$ showed 1C-like characteristics in the vicinity of the destabilizing shear-layer existing the stenosis throat, features that extended further downstream the jet for the Reynolds stresses. For the large-scale energy-containing turbulence (Fig. 3), these findings may be expected as these particular regions are susceptible to high turbulence production due to the strong local mean strain rate. When turbulence moves away from this extreme state, the energy starts to rapidly redistribute and get more three-dimensional. 1C-like turbulence is also expected when the flow starts to relaminarize (Jovanovic et al. 2003), which, e.g., can be seen in certain regions at the LFD phase in this study. The observed anisotropy of the smaller scales associated to the $\epsilon _{ij}$, in the near-wall region and shear layer (Fig. 4), may be explained by the anisotropic nature of TKE production at these sites, which do not favor turbulence isotropization due to the large shear intensity that tends to evoke anisotropy across all scales (Mollicone et al. 2017). This phenomenon is evident in the early stages of the shear layer development (Fig. 4), whereafter the turbulence dissipation attains more isotropic characteristics as the shear intensity weakens and $\epsilon$ increases. In the luminal region, the well-known wall-induced anisotropy layer was found for both these tensors (Liu and Pletcher 2008), where the turbulence states approach the two-component limit. Within this region, turbulence tended to be more axisymmetric, toward the 2C corner, although occasional areas of more 1C-like characteristics were also noticed. The overall magnitudes of $R_{ij}$ and $\epsilon _{ij}$, i.e., TKE and $\epsilon$, respectively, could not be located to one particular region in the AIMs. High TKE levels were mostly found in the vicinity of the jet, as expected, which overall did not show any specific association to any stress-state in the EFD phase. Not surprisingly, high dissipation rates were found along the turbulent spot, with the most extreme intensities in the wake of the jet as well as along the wall impingement region.

It is important to acknowledge some CFD modeling assumptions that may affect the results of this study. However, these limitations are not believed to have a major influence on the general tensor characteristics assessed here but should be carefully considered if more precise quantitative analysis is of interest. Also, this study was limited to one patient-specific case. To assess the generality of these findings, a much larger cohort of patients with varying turbulent-like hemodynamic conditions should be considered. The blood properties were not treated as a dense multiphase suspension of elements, but as a homogeneous continuum (with strain rate dependent viscosity), which is common practice for hemolysis modeling with CFD (Faghih and Sharp 2019; Yu et al. 2017). Red blood cells (RBCs) can act as a barrier that could change the turbulence breakdown behavior for eddies of similar length scales (Antiga and Steinman 2009), which could alter the dissipation tensor characteristics. However, in this study, the estimated resolved scales were an order of magnitude larger than the size of RBCs, suggesting this two-way coupling to be small. The Reynolds stresses are dictated by convective macroscales and will therefore presumably not be influenced by this blood model assumption. The CFD method assumed a periodic flow boundary condition. Here, further patient-specific studies need to be performed to assess the tensors characteristical sensitivity to, e.g., exercise, or slightly varying pulse. Fifty cardiac cycles were used for phase averaging, which previously have been shown to resolve most of the energy-carrying scales of the turbulent flow (Andersson et al. 2015). However, judged by the multitude of less coherent stress-state regions observed for the phase-instant results (Fig. 3a), probably more cycles are needed to converge these tensor characteristics fully. Further modeling assumptions are discussed in previous work (Andersson et al. 2019, 2015) and Sect. 4.3.

### Physiological and clinical relevance

Besides the relevance to assess the general flow accuracy in turbulent flows, which is discussed in Sect. 4.3, these tensor properties may have some direct clinical utility. Parameters related to the Reynolds stresses and dissipation rates have been used extensively to assess turbulence-induced blood damage and significant pressure losses in the bloodstream. These topics will be discussed below in association with the general patient-specific and MRI findings of this work.

Flow-induced blood trauma is a present concern in areas of complex flows where eminent fluid stresses can be expected (Faghih and Sharp 2019), such as at abnormal cardiovascular sites (e.g., various stenosis, unfavorable branching, arteriovenous fistulas/grafts), mechanical heart valves, and blood-transporting devices (e.g., blood pumps). Here, primary attention has been on damage predictions to RBCs (hemolysis), but also to induce harm to the smaller blood constituents (Faghih and Sharp 2019). In turbulent hemodynamics, a wealth of experimental as well as numerical studies has indicated a general increase in hemolytic activity, compared to laminar conditions, while the underlying mechanisms are not well understood and still debated (Faghih and Sharp 2019; Antiga and Steinman 2009; Morshed et al. 2014). Studies have for instance shown that the degree of hemolysis seems to vary substantially for a wide range of different turbulent flows with comparable energy dissipation rates (Bluestein and Mockros 1969), suggesting that the nature of the turbulence characteristics may play an important role. In computational modeling, hemolytic damage to RBCs are typically predicted by a power law (Heuser and Opitz 1980) that is scaled by a fluid scalar stress and various empirical constants, e.g., exposure time (Yu et al. 2017). In turbulent flows, this scalar stresses are usually reduced from the ensemble-averaged viscous stress tensor and Reynolds stress tensor, or a combination of both. In a recent study (Faghih and Sharp 2018), however, it was shown that similar stress levels in different disturbed flows could give rise to three orders of magnitude differences in RBCs membrane tension, findings which question this rather crude hypothesis to represent a complex phenomenon. Here, the authors called for additional, higher-level descriptors of the fluid stress tensor characteristics that would be more universal.

Many researchers have also supported the idea that turbulent fluctuations on the macroscopic scale, represented by the Reynolds stresses, can give rise to sizeable viscous shear stresses at the micro-level (Antiga and Steinman 2009) and result in elevated cell membrane tensions (Faghih and Sharp 2018). These findings have in-part been associated with increased averaged viscous dissipation rates of TKE (Morshed et al. 2014), which in equilibrium flows (where the production and destruction of TKE are balanced) are directly correlated with the magnitude of the viscous shear stress experienced by the cells. However, a recent study indicated an inconsistent scaling between the level of energy dissipation rate and cell membrane tension in different types of flows (Faghih and Sharp 2019), suggesting that this parameter alone is not sufficient for universal hemolysis predictions.

In light of the above, there is arguably a general need for better characterization of the local stress-field (laminar as well as turbulence-related), which hopefully could, at least qualitatively, bridge certain flow phenotypes to blood damage predictions. Part of this journey could be to complement presently used magnitude estimates with, e.g., the local turbulence tensor states described in this work. In turbulent-like hemodynamics, RBCs are expected to encounter a broad spectrum of length and time scales along its trajectory, where consistent exposure to certain conditions may be more susceptible to hemolysis than others. The patient-specific CFD model in this study indicated clear signs of varying turbulence anisotropy characteristics for both tensors ($R_{ij}$ and $\epsilon _{ij}$). These findings are not in-line with the typically simplified view that the turbulence dissipation rate (or small-scale turbulence) can be viewed as being purely isotropic, according to the Kolmogorov hypothesis in fully-developed turbulent flows (Kolmogorov 1991). In fact, the nature of these tensor characteristics is markedly different in the wall-proximity and areas of the turbulence-spot. Here, RBCs may be exposed to a predominately one-component or two-component type of turbulence, which depending on, e.g., the cells concentration, relative motion, and deformation state may interact differently in comparison with a more isotropic environment. This argument is supported by a recent study that showed apparent differences in cell membrane tension concerning the RBCs positioning inside and between rotating eddies (Faghih and Sharp 2018). It is important to note, however, that Reynolds stresses are not real physical stresses. As such, tensor-based descriptors such as the magnitude and anisotropy can only represent a correlation against the actual hemolytic forces on the cellular level. Triple decomposition was used to separate the mean and periodic part of the flow from turbulence-related fluctuations. An appealing extension of the current methods would be to apply a prior frequency-based decomposition on the velocity signal (Khan et al. 2017; Natarajan et al. 2019; Baj et al. 2015), in order to associate different frequency bands to the anisotropy tensor characteristics. Such filtering approaches could also enable the distinction between coherent secondary flow features (shear-layer oscillations, vortex rings/structures, etc.), typically seen in the late flow acceleration phase (Andersson et al. 2019), and turbulence-related characteristics.

MRI-based turbulence measurements have lately been used with the intention to improved noninvasive pressure loss predictions in stenotic flows, from early studies only using normal Reynolds stresses (Dyverfeldt et al. 2013; Ha et al. 2016; Casas et al. 2016) to more recent work that takes advantages of all tensor components to account for the turbulent-related energy dissipation rate (Ha et al. 2017; Gülan et al. 2017; Ha et al. 2019). The latter concept is governed by a dynamic equilibrium assumption between the rate of turbulence production and turbulence dissipation in the investigated domain. Based on these assumptions, 4D Flow MRI has also been used to estimate the turbulent viscous stresses for assessing blood damage (Ha et al. 2016). However, this proof-of-concept study was only investigated on CFD-based MRI simulations in simplified stenosis under steady flow conditions. The robustness of this method in a more patient-specific flow environment remains to be investigated. Also, the validity of the turbulence production-dissipation balance need to be tested, which only occurs if the transport properties (i.e., due to convection, pressure, and viscous diffusion) inside the MRI-voxels are negligible, as well as the limiting low MRI temporal resolution impact on, e.g., the stress accumulation along estimated pathlines. However, before engaging in MRI-based turbulence assessments, it is essential to outline the uncertainty of these tensor parameters. In this study, realistic patterns of the measured TKE, turbulence anisotropy, and stress-states fields were observed (Fig. 7), of which, however, a multitude of voxels, in fact, were physically unrealizable. Robust validation methods of these turbulence-related quantities are hence necessary to underline potential issues with the MRI assessments, which is further discussed in Sect. 4.3.

### Validation aspects

Verification, validation, and uncertainty quantification are required procedures to gain awareness of the errors associated with numerical modeling predictions, which especially applies to blood flow simulations where proper confidence level needs to be reached in order to approach clinical applicability (Steinman and Migliavacca 2018; Berg et al. 2019). In the past decade, a notable rise of turbulence-related hemodynamics studies has been seen, however, with no clear modeling strategy consensus. While plenty of general modeling recommendations have been conveyed for laminar hemodynamics (Taylor and Steinman 2010; Berg et al. 2019; Steinman and Pereira 2019), best practice guidelines related to simulations of patient-specific turbulent-like flows are scarce.

Model validation concerns with the computational model physical representation, i.e., how well does the numerical predictions mimic the physically real conditions (Oberkampf and Trucano 2002), which in the hemodynamic community typically is about comparing results against in vitro or in vivo measurements. However, validation processes could also involve comparisons between different modeling strategies (e.g., choice of boundary conditions, blood treatment, etc.) in relation to a reference model (baseline) that is viewed as a better description of the real conditions. These comparative studies could underpin the relevance of, e.g., less computational expansive modeling assumptions commonly adopted within the biofluid community, such as using steady-state inflow conditions or a RANS turbulence modeling assumption, as showcased in this study. Various versions of RANS-based modeling strategies are still frequently used in image-based computational hemodynamics, despite its long-term discouragement (Ryval et al. 2004; Taylor and Steinman 2010). In our study, the unsteady RANS results showed a clear lack of agreement against the reference LES results (Fig. 6), with near isotropic Reynolds stress characteristics in the entire turbulent region. These findings are in-line with other steady flow studies comparing scale-resolving simulations against two-equations eddy-viscosity models (Emory and Iaccarino 2014). Here, it appears clear that the underlying Boussinesq assumption cannot handle the anisotropic flow features present in these types of flows, and it would therefore be unwise to study any turbulence-related quantities derived from these models.

A common way to reduce computational costs in patient-specific modeling is to ignore the cyclic nature of the flow and instead assume steady inflow boundary conditions, typically based on the pulsatile peak or mean flow rate. In this work, we evaluated the impact on the turbulence tensor characteristics of these flow assumptions against the pulsatile LES model. As both investigated Reynolds numbers showed comparable anisotropic Reynolds stress characteristics in the domain, only the peak flow rate results were evaluated (Fig. 5). In general, a considerable over-prediction of TKE was seen by this assumption, pre and post the CoA region, which was expected due to the overall higher mean flow momentum that induces more energetic and broader turbulence-related energy cascade compared to the pulsatile EFD counter-part. In contrast, the degree of anisotropy showed a generally qualitative agreement between the steady inflow and EFD results, which likely can be associated to the quasi-steady jet observed in both cases. However, by expanding the Reynolds stress tensor to its componentiality representation, it is clear that the steady inflow conditions occupy a much broader spectrum of stress states than the time-averaged pulsatile results. Again, as noted previously, the pulsatile nature (acceleration/retardation) of the flow seems to have a more restricted influence on the turbulence characteristics, which cannot be captured by a steady flows assumption; even considering the relatively small fraction of the cardiac cycles represented by the EFD phase. This temporal constrained characteristics may also shed some light on why ensemble-averaging of turbulence-related quantities, in general, requires several orders of magnitude fewer data samples for pulsatile flows in comparison with non-pulsatile flow in order to attain statistical convergence. To put in perspective, the tensor components for the pulsatile reference case were derived from 50 data samples (cardiac cycles) in this study, while the steady inflow cases required several hundred thousand samples (time-steps).

4D Flow MRI measurements of turbulence properties have been going on for more than a decade, initially by assessing the intra-voxel variance in three normal directions (Dyverfeldt et al. 2006), represented by the TKE, which after that was extended to acquire all six Reynolds stress components using the ICOSA6 scheme (Haraldsson et al. 2015; Kefayati et al. 2015). There are numerous studies that have used these turbulence predictions for in vitro and in vivo investigations, while less attention have been focused on thorough validation procedures, e.g., against well-resolved CFD results. The suggested tensor characterization methods described herein could hopefully aid in the development of more trustworthy predictions.

In this work, the measured Reynolds stresses for the two flow cases showed expected jet-flow as well as TKE characteristics in the post-orifice region (Fig. 7). However, the more in-depth analysis by anisotropy mapping indicated nonphysical properties in parts of the most turbulence intense regions. These unrealizable predictions would be more expected in less turbulent-prone regions due to reduced signal-to-noise ratio (SNR) and wall interference. However, it is important to note that voxels that satisfy the realizability constraints do not necessarily imply that they are physically correct within the AIM. For example, it is not reasonable to expect that the highest TKE levels to only be found in the near-proximity of the 1C corner of the AIM, as seen in the MRI-predictions (Fig. 7), as this extreme stress-state normally is evident in areas of maximum turbulence production (Gorlé et al. 2012) and relaminarizing flow regions (Jovanovic et al. 2003). Furthermore, these characteristics were not seen to the same extent in the patient-specific steady flow results (Fig. 5c), supporting the argument that the MRI-based 1C-like characteristics may be erroneous in these flow cases. However, this remains to be determined by validation against representable CFD results. These tools could also bring value into the sensitivity analysis of the 4D Flow MRI acquisition parameters itself, e.g., to outline the characteristical changes of the Reynolds stress tensor due to particular measurement settings (velocity encoding parameter, spatial and temporal resolution, SNR, acquisition time, etc.) as well as the influence of post-measurement data corrections (e.g., due to background phase errors, partial volume effects, higher-order motion, etc.).

### Conclusions

This work has demonstrated an efficient approach on how barycentric anisotropy invariant mapping can be used to characterize ensemble-averaged turbulence-related tensors (such as Reynolds stresses and dissipation rates) in patient-specific cardiovascular flows as well as MRI measurements, while also reflecting on the methods potential relevance in blood damage predictions. In the patient-specific CFD model, the suggested approach uncovered a broad spectrum of turbulence anisotropy characteristics throughout the flow deceleration phase, with partly coherent turbulence states at several sites in the post-stenotic region. However, the transient nature of some of the more extreme states appeared to be short-lived. The generality of these findings, however, needs to be confirmed over more patient-specific flows and cardiac cycles. We also presented how the turbulence tensor states derived from this approach can be a practical and comprehensive way to evaluate the credibility/accuracy of CFD as well as MRI-based turbulence data. If tensor-related turbulence quantities are of primary concern, findings in this study discourage using a steady inflow assumption over pulsatile conditions and two-equation RANS over LES models. Qualitatively, the MRI-based turbulence measurements of the two flows showed overall expected TKE, anisotropy, and stress-state patterns. However, the proposed method could also detect voxels with nonphysical or possible unrealistic turbulence characteristics, even associated with the $25\%$ highest TKE levels. These findings suggest that more detailed studies of MRI-measured turbulence fields need to be taken, which hopefully can be facilitated with more comprehensive evaluation tools as showcased in this study.

## Footnotes

1 Corresponding to the number of anisotropy eigenvalues $\lambda _{i}\ne -1/3$ and represent the tensor rank, or number of principal axes with active turbulent fluctuations.
